# Are return-of-service bursaries an effective investment to build health workforce capacity? A qualitative study of key South African policymakers

**DOI:** 10.1371/journal.pgph.0000309

**Published:** 2022-05-05

**Authors:** Sikhumbuzo A. Mabunda, Andrea Durbach, Wezile W. Chitha, Blake Angell, Rohina Joshi

**Affiliations:** 1 School of Population Health, University of New South Wales, Sydney, Australia; 2 The George Institute for Global Health, University of New South Wales, Sydney, Australia; 3 Australian Human Rights Institute, University of New South Wales, Sydney, Australia; 4 Health Systems Enablement and Innovation Unit, University of The Witwatersrand, Johannesburg, South Africa; 5 Institute for Global Health, University College London, London, United Kingdom; 6 The George Institute for Global Health India, New Delhi, India; University of Birmingham, UNITED KINGDOM

## Abstract

Return-of-service schemes (RoS) or bursaries are used in South Africa and other nations to publicly fund the training of skilled health professionals in return for the beneficiary agreeing to return to serve in their local provinces on a year-for-year basis. This study aimed to understand insights of key policymakers in South African provinces to identify barriers and solutions to implementation of RoS schemes used to recruit and retain skilled health professionals. This research draws on the insights of 16 key South African policymakers from eight of its nine provinces through semi-structured, qualitative interviews. The respondents were interviewed through Microsoft Teams virtual platform, either in pairs (ten) or as individuals (six). Data were analysed using inductive, thematic analysis in NVIVO. The study was reported according to the consolidated criteria for reporting qualitative research. Respondents reported that the schemes had resulted in an increase in the number of skilled health professionals and had provided opportunities for study and employment for previously marginalised groups. Formal evaluations of the impact of the schemes were not reported, however, a number of shortcomings with current schemes were identified that were likely limiting their effectiveness. Respondents reported a lack of foresight in the scheme implementation including a bias in the selection of beneficiaries towards medical professionals at the expense of other health workers. Furthermore, failure to plan for practice location when beneficiaries finished training limited the capacity of the schemes to meet the needs of local populations. Monitoring of recipients was limited by loopholes in contract design, decision-making and poor coordination between departments. Between 1 and 30% of beneficiaries were reported to default their contracts with some not completing their studies, some not returning after completing their internship and others terminating their services before concluding their contracts. Return-of-service schemes have helped in overcoming health professional shortages. However, they haven’t been formally evaluated. Several planning and implementation shortcomings were identified which can be improved to enhance access to healthcare in South Africa.

## Background

Shortage of skilled health professionals is a major barrier to universal health coverage, especially in low- and middle-income countries (LMICs) [1]. These shortages result from underproduction, maldistribution to either urban centres and/or the private sector, and emigration to high-income countries [2–4]. For instance, South Africa’s public health sector has 0.64 anaesthetists per 100,00 population, compared to 9.69 in the private health sector whereas the country only needs 5 per 100,000 population [5]. The country’s public health sector also only has 33 per 100,000 medical practitioners compared to a global average of 176 per 100,000 population [5–7]. Similarly, public sector primary care facilities are only serviced by 29.1% of the required professional nurses and midwives [5]. Addressing this shortfall requires substantial and strategic investments in the health workforce, as well as improvements in health workforce-related planning, education, deployment, retention, management and remuneration [1–3, 8–13].

South Africa has for years invested in education of health sciences students to increase the number of health professionals and improve their retention through government sponsored or non-government organisation (NGO) sponsored return-of-service (RoS) schemes [4, 5, 13]. Health sciences students are funded to study either locally or for some medical students, internationally, e.g. Cuba with an agreement that after their training, they will return to serve their provinces for a specified period of time [13, 14]. Some NGO RoS schemes like the Umthombo Youth Development Foundation [6, 15] and the Friends of Mosvold Scholarship scheme [16, 17] have previously been reported on in more detail and found the schemes to have a good return on investment [6, 15–17]. However, to date, there is little literature scrutinising the challenges, implementation process and sustainability of the government sponsored schemes [4, 13, 16–21]. This study seeks to fill this gap through an examination of insights from key policymakers in South African provincial health departments on how they have designed and implemented health professional training policies, and to identify barriers and solutions to implementation.

### Research context

This research was conducted in eight out of nine South African provinces. As South Africa has a devolved system of governance, provinces are responsible for the provision of healthcare through their respective Provincial Department of Health (PDoH) as mandated by the Constitution [22] and the National Health Act [23]. They employ health workers under conditions set out by the national Department of Public Services and Administration. Each PDoH derives its own recruitment, training and retention strategy to meet required health needs.

### Reflexivity and positionality of the researchers

Both SAM, a public health medicine specialist and WWC, a health economist are Black South African males with a rural upbringing who each benefited from a RoS of two different participating provinces for their undergraduate medical studies. As undergraduate medical students, both participated in rural elective placements and the executive structures of a student run organisation known as Rural Support Network [24]. They have over 10-years’ experience in the South African health system as clinicians, managers, researchers and academics, and through this have known some of the participants in a professional capacity prior to this study. AD is a South African born academic and lawyer with a special interest on human rights advocacy. BA is a health economist researcher with experience examining health systems and policies in countries of all income levels. RJ is a global health workforce leader who has for most of the last decade, dedicated her life to the finding of sustainable health workforce solutions in LMICs.

## Methods

### Design

Using qualitative critical theory methodology, we designed the study to enable an assessment and critique of the current situation (i.e. attempting to overcome shortages of health professionals in underserved communities) [25–28]. This strategy allows for assessment of the challenges in the implementation of RoS schemes and thus derive solutions to overcome them.

### Sample and recruitment

We aimed to interview the entire initially identified population [19] of South policymakers from nine provinces. Considering the limited size of this population, both convenience and snowball sampling were used. Participants were purposively identified by referral through the office of the accounting officer in each province and department. Through snowball sampling we identified a key policymaker from one Provincial Department of Education. Participants are individuals who were directly involved with the development and implementation of health related RoS schemes in government departments of all nine South African provinces. Inclusion criteria were as follows: government officials managing bursaries and responsible for the formulation and monitoring of RoS policies, for budgeting bursary policies or involved with the deployment of RoS beneficiaries. Officials were excluded if employed for less than two years in this role. This resulted in the exclusion of one province. Recruitment occurred through email advertisements in the PDoH offices. Four participants were excluded because one participant was new in the position and three could not be reached.

### Data collection

Due to COVID-19 travel restrictions and additional workload for participants, Microsoft Teams was used for interviews at times that suited participants over a period that spanned January and October 2021. Participants were in their workplaces during the interviews which took an average of 75 minutes. Potential participants were given verbal and written information on the purpose of the study at least two weeks before the interviews. We used interviewer guide-aided open-ended interviews (S1 Interview guide) and summarised in Table 1 to understand the formulation and implementation of RoS policies. The interview guide was developed using the researchers’ knowledge of the process and identified literature gaps. Six participants were interviewed as individuals, and ten were interviewed in pairs. All eleven interviews were conducted by the first author (SAM) to ensure consistency. The initial transcription was obtained from Microsoft Teams, which was transferred to Microsoft Word and cleaned by the first author who checked all transcripts against the recordings and translated the non-English language phrases used in the interviews (from a total of five languages) to ensure that the transcriptions were verbatim.

**Table 1:** Abridged interview guide.

No.	Area of Interest/topic
1.	Origins and evolution of the policy^¥^
2.	Custodian of the policy
3.	Review of the policy
4.	Decision process
5.	Contract
6.	Process after the completion of studies
7.	Policy Challenges
8.	Monitoring and evaluation of the policy

^¥^Not discussed in this article

### Data analysis

Data were analysed manually using inductive, thematic analysis in NVIVO 12 (S1 Codebook). The first author reviewed all the transcripts, the fourth and fifth authors independently reviewed half of the transcripts. The three researchers discussed their independently developed codes, and the first author synthesised the results. The analysis followed a six-step approach of familiarisation (reading and re-reading transcripts making observational notes), coding (import transcripts into NVIVO and systematically breaking down responses into broad interpretations based on the research question), theme development (constructive process of developing themes), review of themes (co-authors reviewed themes), defining themes (revisited and finalised themes) and reporting as advised by literature [29]. The results are presented using five themes and five sub-themes that emerged from the data to answer our questions.

### Ethics approval

Ethics approval was granted by the Human Research Ethics Committees of the University of New South Wales (HC200519) and the Walter Sisulu University, South Africa (065/2020). Research access approval was obtained from all nine Provincial Health Research Committees in South Africa. All participants gave their written informed consent to the audio-recording of interviews through Microsoft Teams. Participant names and provinces were de-identified.

### Inclusivity in global research

Additional information regarding the ethical, cultural, and scientific considerations specific to inclusivity in global research is included in S1 Checklist.

## Results

Sixteen out of twenty policymakers from eight provinces were interviewed for this research. Females accounted for 7/16 of respondents. Respondents’ experience of managing RoS schemes at provincial level ranged from 5 to 23 years. Fifteen respondents were from the PDoH and one was from the Provincial Department of Education.

Participants offered their insights on the purpose, return on investment, factors affecting effectiveness, unintended consequences and sustainability of RoS schemes in their respective provinces.

### What are return-of-service schemes?

Bursaries, as RoS schemes are known in South Africa, are used to fund the study of beneficiaries on the understanding that they will serve health facilities which find it difficult to recruit health professionals for at least the same number of years as the candidate was funded during their studies. Whilst most beneficiaries are funded for the duration of their studies (average of 6-years for medicine if studying in South Africa, 9-years for medicine if studying in Cuba and 4 years for most other qualifications), some beneficiaries can be funded for less than that, e.g., 1-year if only offered the sponsorship in their final year of study. As one respondent described:

…will pay for them for the amount of years (sic) that they need to go study, for example, if it’s four years or six years in medicine (sic)… and at the end of the qualification they can work for the Department of Health… they don’t pay us back any money, they come back and work for the department and they will still get their full salary.……we need their services in our hospitals.

RoS Schemes are managed in each province by the bursary office through the human resource development office. In five of the provinces (Eastern Cape, Gauteng, KwaZulu-Natal, Limpopo and Western Cape), the policy is entirely managed directly by the Department of Health. Funding for study within the country has been shifted to the Office of the Premier in the North West province and to the Department of Education (DoE) in Mpumalanga and Northern Cape provinces. In addition to local universities, all provinces, except for the Western Cape (WC), send students to Cuba for medical studies. The PDoH is responsible for funding and recruitment of students studying in Cuba. Mpumalanga province (MP) also sends students to Russia for medical studies, who are funded by the DoE. Clinical professions trained can include but are not limited to medicine, physiotherapy, nursing, radiography, medical specialty, nursing specialty, etc. Box 1 summarises the themes that emerged from the interviews.

**Box 1. Summary box on return-of-service schemes in selected South African provinces**

**Table:** 

Theme	Example
Underlying objectives of return-of-service schemes.	“…the… Province is a rural province… …it was difficult to recruit health professionals to serve in our rural areas”.
	“…we thought that … the bursary system will assist to be able to get those skills … that we don’t have as a province…”
	“…the bursary came in as a means of ensuring that… we always have a constant supply of skills, in various Health Sciences fields”.
Return on investment of RoS schemes.	…we had 59 million [Rands (~4.2 million USD)] (*spent on bursaries*) in 2016/17, then it went to 61 million [Rands (~4.4 million USD)], … 2019 …it was 212 [million Rands (~15.1 million USD)] and then last year it was 228 million [Rands (~16.3 million USD)], and then this year it is even doubled, so it’s a lot of money. “…
	“No, I don’t know any formal evaluation”.
	“We are short of those researchers who are serious. …somebody.… must evaluate your work”.“…the only evaluation that we do now is when we speak to the students, four times a year, quarterly, and that’s the only evaluation that we do at the moment”.
What factors affect effectiveness of the policy?	“…it’s very difficult for another department to plan on our behalf”.
	“…it just says you will work back. It doesn’t say where you will work back or that you’re going to be linked to where you come from or something like that”.
	“…it really pains us a lot. …when a person just leaves just like that and some of them are quite intransigent… one was claiming marriage. …to say that the boyfriend is from (*another province*) …”
	“…after you’ve allocated these people pop up and they say: ‘But I’m a bursary holder…’, and they are nowhere on the list to be found”.
	“But now the problem is once these children (*beneficiaries*) go to university they will come back, and they will tell you: ‘But my circumstances have changed. I’ve now got married’…now we want to be humane and we want to accommodate these people and that sort of makes it to me…why do we give a bursary?”
	“…both of them just went and they took out a loan and paid back the amount which they got for the bursary. Because remember that is an interest free loan.…there is no way… what they pay to us and what we lose…. because now like I said, those two doctors paid each 350,000 Rands (~25,000 USD*) …”
	“…there are instances where you don’t need a doctor, but you need a physiotherapist…we have scarcity. You need a psychologist, we have scarcity. So, there are those professions that… are also as important as having a medical practitioner but, because I think the view is that you know if there is a doctor, it can address a number of conditions…”
Unintended consequences	“I have got now children (*graduate health professionals*) that studied with actual loans from banks. Which need to pay it back and that loan every year accumulate interest. And that child actually wants a job, but we now are prioritising these ones. It doesn’t matter if they are good or bad. We prioritise them and they don’t even want to come back”.
	“We’ve got so many children (*health professionals*) that’s unemployed”.
Sustainability	“…we were just verbally informed to say that look guys, the Department is going through some challenges and because of cost containment and then austerity measures that we were going through please don’t again advertise (*sic*)”.
	“…if we are going to shrink the budget and not putting those youngsters that want to further their studies…they are going to have a revolution. So, the country is going to become unmanageable”.
*Exchange rate used is 1 USD = 14 South African Rands

### Return on investment of RoS schemes

All provinces reported a positive impact of RoS schemes on training health professionals to deal with the shortages and maldistribution of the health workforce in South Africa. Some also highlighted the social effect of the bursaries for people who otherwise would not have been able to obtain a qualification.

…to support… disadvantaged children from… disadvantaged families.……Especially those who pass very well in matric.

#### Lack of evaluation of RoS schemes

While all the provinces contact the students while they are being supported by the bursary, none of the schemes have been systematically evaluated. Respondents also noted the substantial cost of the schemes and specific instances of waste.

… nobody comes back at the end of the year and say: ‘Ok, we have generated so many bursaries, how much did it reduce your vacancy rate? What was the impact of this’?

The respondents suggested that it was important to review the strategy for workforce planning and deployment, and utilisation of resources:

…it’s time that we… approached our implementation… of plans and utilisation of resources using scientific approaches because many at times we have caught ourselves…, finding that we have been implementing wrong decisions. All of a sudden maybe there is oversupply, or interestingly enough…… we find that the reason why we do not have medical practitioners in our facilities is not (*sic*) that we do not train them, but it’s because we are unable to retain them.

#### Contract breach equates to loss of skilled health workforce

All provinces experienced defaulters or individuals who breached their contracts, and this was estimated to range from 1–30% of funded individuals. One province also estimated that about 20% of the defaulters would not have served at all, and only half of the defaulters will settle their debt. Even if money is to be repaid, it’s not an easy exercise to quantify and/or replace their loss as suggested by a participant from a rural province:

But we lost a whole doctor!!! Two of them. Because it takes us another six years to produce… Seven…six…seven…eight years to produce a doctor!!!

Even though the two urban provinces (Gauteng and Western Cape) also reported defaulters, this did not seem to impact them as negatively, hence they could be able to say that: “We are still fine, we are working fine for few years now (*sic*), luckily”.

#### What factors impact effectiveness of the policy?

Respondents noted that, while South Africa has shortages of many groups of health workers, bursaries were often skewed towards medical doctors without any assessment of the skills-mix needed in a provincial health system based on assumptions.

Even the… international arrangements that I have mentioned, the Health Sciences students that we send there is medical practitioners and nothing else. So, that on its own will tell you that it looks like the bias is on medical practitioners.

Most of the health professional beneficiaries complete their studies because they are “…a group of serious cadres who really go out and pass”. However, poor academic progression by a minority, impacts effectiveness of the schemes as not everyone who is funded is able to complete their studies. While the contract mandates the student to pay back the funding if they fail to complete the course, the participants felt that it was challenging to enforce the contractual clause.

According to the contract, the person must pay back the money. Though it’s very difficult to say to somebody… who is coming from a poor background to say: ‘pay the money’. While the person is failing even… to finish his studies. Where is he going to get the money? That is a challenge which is not easy to practice.

Despite the absence of an opt-out clause in at-least one provincial contract, some beneficiaries choose to opt-out. The opt-out clause gives beneficiaries the option to re-imburse government a pro-rata amount upon early termination of their contracts.

#### Lack of data integration

In some provinces, the bursaries are distributed by the Department of Education or Office of the Premier and the jobs are provided by the Department of Health, consequently, the data between the two departments is not integrated. Complexities introduced through splitting responsibility across departments include administrative bungles where the bursary holder is not known due to incomplete records at the funding department, in provinces where the Department of Health is not in control of the entire process. This poor communication and coordination leads to poor effectiveness of the schemes.

…they also give bursaries for things (*qualifications*) which doesn’t (*sic*) exist and then the person comes back, and the person say (*sic*): ‘…I must be employed, I am a bursary holder’. … But the person is nowhere registered and no Council (*health professionals’ regulatory council*). It’s not a health profession but it’s a nutritionist. He needs to study another three years whilst we employ him. So, now it’s our problem.

Another challenge “…is to get people to work where you actually need them”, thus needing to strike a balance between the preferences of the individual and the needs of the health system. Urban, peri-urban, and rural health facilities that are in a provincial boundary with urban centres are the most popular choices among beneficiaries.

…it’s a very small hospital… It’s close enough to Pretoria for you… to do your private practice in Pretoria. …they are rural hospitals. …you get a rural allowance… and then they don’t pitch up to the hospitals, and they have their private … surgeries (*practices*) in town.

In some instances, there are different interpretations of contractual terms as the contract was ambiguous and beneficiaries did not know where they would be placed to serve the contractual period. The bursary contract also doesn’t specify the community that the beneficiary will be linked to on completion of their studies, despite this being the stated purpose of having RoS schemes.

I remember with the Cuba one, for example, the initial batch, signed the contract, which was designed by national and at that time the contract was blindly saying: ‘You are expected to come back and serve in the country’.

In other instances, the contracts may be open to different interpretations which can lead to possibilities for abuse:

We consulted some… learned lawyers and they told us: ‘No, there is no way you will win this one in court’.

#### Systemic challenges

Other challenges included using paper-based records and non-sophisticated excel spreadsheets to track beneficiaries some of which have been inaccurate in the past. In addition, some departments were reported to have lost some records and have a poor monitoring system. Even though the database of all the provinces is electronic now, the contracts are still paper based which makes monitoring the system cumbersome. Other systemic challenges such as corruption and maladministration have created barriers in the implementation of the program. For example, there have been instances where students from rich families who were not eligible for the RoS scheme have benefitted from the educational initiatives.

You know friends will be just walking to the office: ‘I want a bursary’ and it’s given. When you look at the list of bursars compared to the ones… approved, it was a different number.

#### Changing individual circumstances

Sometimes, beneficiaries of the educational bursaries have not been able to fulfil their contractual agreements due to changing personal circumstances and ambitions.

‘I might come from a poor village, but I’m not destined to be poor, or I’m not destined to live in a poor village’. … you already are acclimatised to urban life. If you have already started a family, your children are attending some, well, fancy…, well-developed schools there, and you know that ‘if I go back to my province, there’s a huge likelihood that they will put me in a rural facility’ and then all of a sudden you lose all the privileges of being in an urban area. So, you will find that the bursary holders will always try to find a way of not coming back…

However, the policy implementors believe that beneficiaries should fulfil their obligations regardless of the changing circumstances as highlighted by a respondent:

‘I have now got children and my husband is here or my wife is here. I can’t go there anymore because I have all these responsibilities’. …but those responsibilities are secondary because your first responsibility was your bursary.

Notwithstanding, there are structural constraints of the departments including lack of expertise for health workforce planning.

we are not having qualified HR (*Human Resource*) practitioners…

### Unintended consequences

Government bursaries come with the advantage of guaranteeing a government job for beneficiaries. However, at times this is to the disadvantage of non-bursary beneficiaries, some of whom would have studied using bank loans. As one respondent said:

…in these years, three years now, we do not take the non-bursary holders at all because we don’t have budget (*sic*).

Some defaulting RoS beneficiaries have tried to return to the public health sector after being dismissed for misconduct in the private sector:

Then you’ve got the other side of the coin; the person leaves, she gets fired outside and then she wants to return, which we are not allowing. We don’t allow you to return once you’ve gone because you make trouble outside. Then you want to come to our patients which can’t choose a doctor

Another challenge is that the obligatory period of service could delay specialisation and professional development for those who have such intentions. For some beneficiaries, this obligatory period could be as long as eight or more years for medical students who studied in Cuba.

### Sustainability

Poor economic conditions have led to conflict between a prior government undertaking to employ beneficiaries post-completion of their studies and the need to finance these posts. This in turn has led to a lack of funded posts for RoS graduates in some situations.

…it was sustainable but now …with this sudden change… with… issue of funding…we are not even sure whether this section is going to (*laughs*)… to be here for a long time. Because now…like even Cuba, we stopped sending the students to Cubans (*sic*)…. We sent ten in 2015 and then we stopped from there.

We actually cannot even afford to appoint them because we still have a lot sitting at home unemployed.

There has also been a recent change in policy to support the education of economically disadvantaged students throughout the country. All students who are assessed as being poor, now qualify for free tertiary education through support from the National Students Financial Aid Scheme (NSFAS), in a way rendering the RoS schemes redundant for poor students.

…the budget which was used for bursaries, were taken from the department… and… were given to the tertiary institutions to strengthen up the NSFAS (*National Students Financial Aid Scheme*). So, the minister of finance, view it: ‘If you are health, let us deal with health issues…let Minister of Higher Education deal with development of skills’…

However, NSFAS funding also has a quota of students that it can fund in a year. This inevitably leads to the exclusion of other needy students who have academic potential. Hence one respondent could confidently say of government bursaries:

…I don’t see this programme discontinuing anytime soon.

## Discussion

In this study, policymakers highlighted the role of RoS schemes in increasing the pool of skilled health professionals. They indicated that RoS schemes improved the prospects of rural and disadvantaged South Africans in securing academic qualifications and jobs in the health sector. These benefits, however, were undermined by lack of long-term planning, lack of transparency in the selection of some recipients, poor monitoring and coordination, and deliberate breaches of contract. No formal evaluations of the impact of RoS schemes was reported despite the high costs of bursaries, which, for example, were reported to account for 25% of the health budget devoted to health worker training in Gauteng province, a significant expenditure in a resource constrained health system [30].

Various factors discouraged beneficiaries from serving out their contracts. These include structural and systemic challenges such as lack of collaboration between departments, maladministration or outright corrupt practices, poor record keeping, lack of specificity in the contracts and poor planning and inadequate monitoring of the scheme [20, 21]. Poor intersectoral collaboration between the departments of health, education and Office of the Premier was found to result in incomplete records and financial waste [10]. Close collaboration between health planners and funders is essential if RoS schemes are to be a reliable health workforce planning tool. Similarly, systems to allow close collaboration and communication between these groups need to be developed to overcome these shortcomings. Delineating roles and responsibilities between different departments will also reduce the complexity of the operation of these schemes. RoS schemes were found to lack strategic planning and did not consider the ideal skills-mix required to address the health needs of the population nor the economic realities of resource-constrained health systems [8–10]. Beneficiaries were found to not only serve their obligations in underserved communities but also in facilities servicing affluent communities undermining the credibility of the schemes which are supposed to benefit disadvantaged areas.

While the rural origin medical students targeted by these RoS schemes have previously been found to be three times more likely to return to rural practice [2, 3, 11, 31–35], a large number were found to prefer urban job placements following their training. Personal circumstances were found to often change during training, as beneficiaries could find a spouse who might not be able to relocate with them due to their personal commitments including RoS obligations with another province. The impact of defaulting on contracts (ranging between 1–30% of bursary recipients) was felt differentially across the provinces. Urbanised provinces which had access to medical schools did not seem to be as negatively impacted by beneficiaries who defaulted their contracts. On the other hand, less urbanised provinces highlighted that losing even one health professional had large implications for the health system. This, therefore, shows that whilst RoS schemes might not be needed in urban provinces, they are essential for less urbanised provinces and need to be enhanced and restructured. Across all provinces though, defaulting beneficiaries represent a significant threat to the operation and impact of RoS schemes. Poor implementation of the scheme is partially enabled by structural issues (e.g. unqualified human resource for health staff) which needs strengthening to enable beneficiaries to serve their obligations.

RoS schemes could be enhanced with evidence-based planning of health workforce, better monitoring and enforcing the fines when contracts are broken. Policies to better support beneficiaries to fulfil their contracts may also improve the functioning, effectiveness, and value of these schemes. Initiatives to include flexible arrangements between provinces to allow exchanges or sabbaticals where they pay back their debt serving another underserved community, for example, might improve retention and health system functioning and should be further explored.

**Fig 1:**
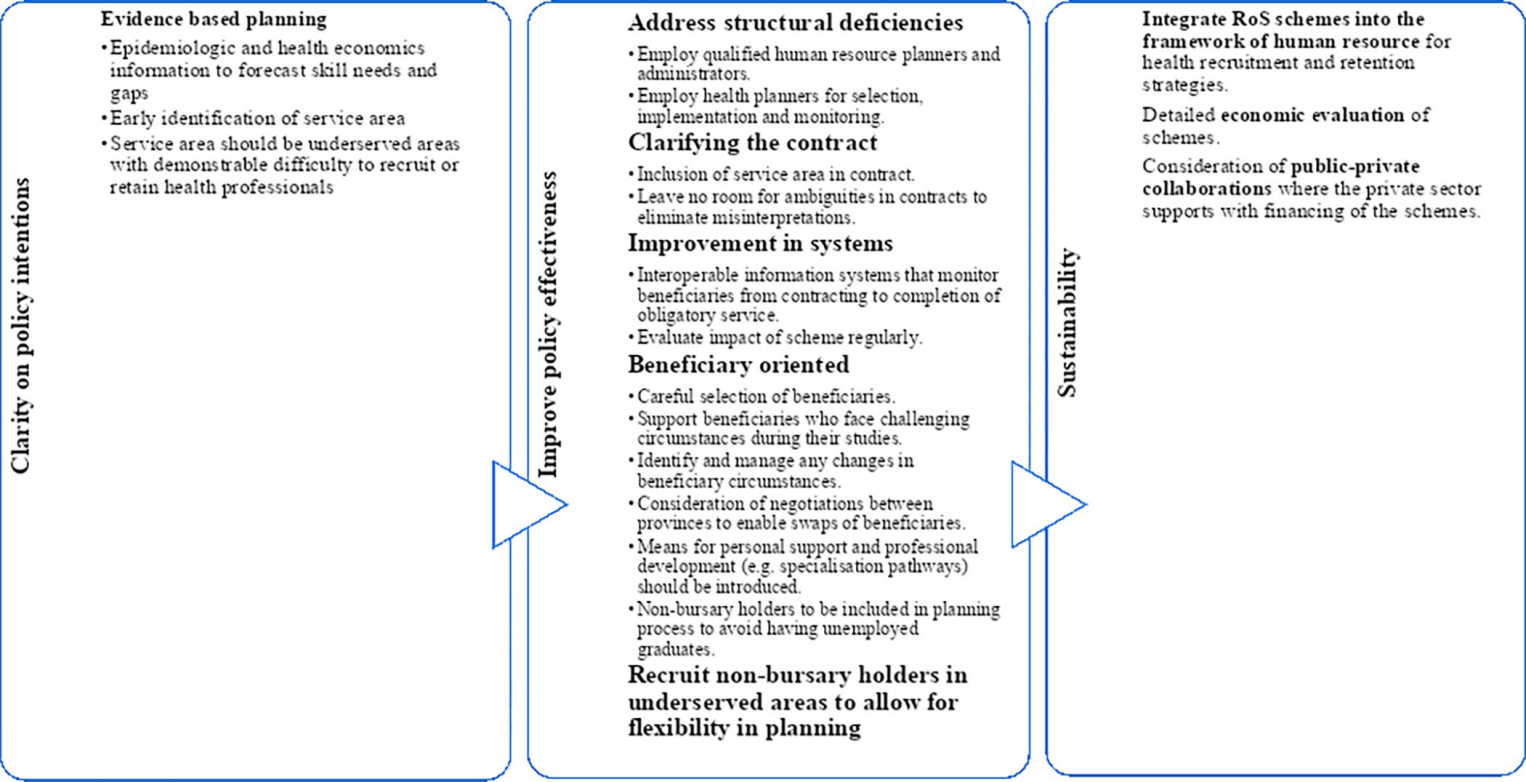
Policy implications for return-of-service schemes.

Though minimal, variation strategies should be considered for beneficiaries who fail to complete their studies. Such strategies could include more careful selection of beneficiaries, proactive personal and extra academic support, negotiations with local tertiary institutions to consider the beneficiaries for another health sciences programme to minimise financial waste. If all else fails, forgiving the debt could possibly work out better for all parties. Fig 1 summarises policy implications of the programme. While our work was conducted in South Africa, similar programs operate across the world. Our findings may help to inform the operation of these schemes, while they too may provide important lessons to improve for the South African schemes studied here. In Australia, for example, RoS beneficiaries of the Medical Rural Bonded Scholarships scheme are able to reduce their obligatory period by serving remote areas [36–38]. Changes affecting beneficiaries are also discussed by beneficiaries and health planners [36–38]. Similarly, initiatives to encourage beneficiaries to serve their obligations used in these other programs may offer insights into ways to overcome the problems raised in this study to improve the functioning of these schemes in South Africa [36–38]. Incentives such as priority school admissions of their children and low interest rate loans have been used in Sri Lanka where 87% of medical specialists trained were found to serve their obligatory period [39], while better clarity or agreement with the beneficiary on where they will serve have been used in India and Canada to improve retention [20, 39, 40].

### Strengths and limitations

Our study has some limitations. We interviewed stakeholders from eight of South Africa’s nine provinces. While the excluded province is not systematically different from those included in the study, there may have been some distinctions which were not captured. Second, whilst we ensured the inclusion of all the key policymakers across the eight provinces, unfortunately, we missed some representatives from the DoE and Office of the Premier and Human Resource management in some of the provinces.

## Conclusions

Return-of-service schemes have been used by South African provinces with the aim of building health capacity in underserved areas. These schemes have been socially responsive in helping people from disadvantaged backgrounds who would not have been able to obtain an educational qualification in the health sector without the bursaries. They have helped in overcoming health professional shortages. However, these policies are often poorly planned, coordinated and monitored and have not been formally evaluated. They have at times been implemented with opportunity costs of not employing other deserving citizens and at times employing incompetent beneficiaries, thus rendering them non-sustainable. To have a lasting impact, health workforce planning strategies need to be non-sporadic, long-term and continuous in nature, with ongoing monitoring and evaluation to ensure they are contributing to these objectives and guard against unintended consequences. Planning and managing a successful RoS scheme should also be based on current and forecast health needs, sufficient allocation of resources for future salary needs, efficient monitoring mechanisms to ensure compliance and the integrity of beneficiaries and policy custodians.

## Supporting information

S1 Checklist COREQ (COnsolidated criteria for REporting Qualitative research) checklist. (PDF)

S1 Codebook (DOCX)

S1 Interview guide (DOCX)
