# Supplementary material for: Are return-of-service bursaries an effective investment to build health workforce capacity? A qualitative study of key South African policymakers
Source: PLOS Glob Public Health. 2022 May 5;2(5):e0000309. doi: 10.1371/journal.pgph.0000309 (PMC10021585; doi:10.1371/journal.pgph.0000309)
Supplement: S1 Codebook — (DOCX) [file pgph.0000309.s002.docx]

Codebook

Nodes

| Name | Files | References |
| --- | --- | --- |
| Effectiveness and value for money | 2 | 4 |
| Impact of program | 5 | 13 |
| Benefits to individual | 1 | 1 |
| Other benefits to individual such as a job or opportunity to travel | 0 | 0 |
| Trained when otherwise wouldn’t have had the chance | 0 | 0 |
| Benefits to system | 3 | 4 |
| Health worker retained | 0 | 0 |
| Health worker trained | 0 | 0 |
| Health workers directed to underserved areas | 0 | 0 |
| Other benefits | 0 | 0 |
| Shortcomings of the program | 0 | 0 |
| Conflicting clauses | 1 | 1 |
| Timing of health workers ‘loss’ to the system | 7 | 28 |
| Unintended negative consequences | 5 | 32 |
| Programme evaluation | 10 | 22 |
| ROS Challenges | 4 | 5 |
| Audit Compliance | 5 | 12 |
| Beneficiary misrepresentation | 2 | 2 |
| Changing personal circumstances | 5 | 14 |
| Contract validity questionable | 1 | 1 |
| Coordination | 11 | 73 |
| Corruption and maladministration | 8 | 23 |
| Defaulters | 10 | 113 |
| Swap deal | 4 | 10 |
| Increased production of health professionals | 3 | 13 |
| Information systems | 8 | 31 |
| Poor Information Systems | 8 | 43 |
| Lack of a signed policy | 1 | 4 |
| Lack of Funding | 8 | 38 |
| Bottlenecks and inefficiencies | 6 | 26 |
| Early Release | 10 | 20 |
| Grant Terms | 8 | 27 |
| Limited Career Development Opportunities | 1 | 5 |
| Poor Academic Progression | 6 | 14 |
| Poor Skills-mix | 6 | 9 |
| Research Paucity | 9 | 34 |
| Resource availability | 5 | 7 |
| RoS Loopholes | 8 | 23 |
| Salary forecast | 8 | 19 |
| Saturation | 4 | 7 |
| Unhappy beneficiaries | 3 | 4 |
| Urban-based Medical school | 2 | 4 |
| How they operate in practice | 0 | 0 |
| Best Practices | 5 | 11 |
| Certainty | 7 | 27 |
| Contract details | 8 | 65 |
| Beneficiary responsibility | 7 | 44 |
| Contract Variation | 5 | 12 |
| Custodians' responsibility | 6 | 13 |
| Data Credibility | 4 | 11 |
| Decision process | 11 | 196 |
| Political decisions | 7 | 20 |
| Monitoring during studies | 1 | 3 |
| Monitoring post education | 4 | 9 |
| Are bursary holders followed up | 6 | 15 |
| Minimum education standards that must be met to retain bursary | 5 | 8 |
| Sanctions for breaking contract | 9 | 58 |
| Needs Determination | 11 | 131 |
| Poor Communication | 5 | 19 |
| Recruitment to service | 8 | 58 |
| Special circumstances for placement | 2 | 4 |
| ROS Prescription period | 2 | 2 |
| Service Placement | 10 | 56 |
| Guaranteed | 5 | 18 |
| Not guaranteed | 6 | 10 |
| Placement planning | 8 | 49 |
| Service placement non-negotiable | 3 | 10 |
| Service termination | 3 | 3 |
| Leaving to be with friends or family | 2 | 5 |
| Stakeholders | 6 | 72 |
| Training countries | 9 | 41 |
| Unemployed beneficiaries | 2 | 4 |
| Justification | 1 | 1 |
| Reason for ROS | 0 | 0 |
| Aim of program | 10 | 37 |
| Disease burden | 1 | 1 |
| Retention | 5 | 7 |
| Contract details | 8 | 62 |
| Beneficiary responsibility | 6 | 39 |
| Contract Variation | 5 | 11 |
| Custodians' responsibility | 5 | 10 |
| Previous policy shortcomings | 1 | 1 |
| Problem policy is looking to overcome | 0 | 0 |
| Policy Background | 3 | 28 |
| Operational guidance | 1 | 5 |
| Legislation | 5 | 22 |
| Pillars | 1 | 1 |
| Capacity Building and development | 8 | 39 |
| Beneficiary characteristics | 6 | 23 |
| Entitlement | 1 | 8 |
| External Bursary | 8 | 112 |
| Gratitude | 1 | 1 |
| Internal Bursary | 3 | 3 |
| Middle class beneficiaries | 2 | 4 |
| Missing middle | 3 | 8 |
| Rural origin beneficiaries | 2 | 5 |
| Sense of belonging | 1 | 1 |
| Effectiveness | 2 | 20 |
| Contentment | 2 | 7 |
| Ownership | 4 | 21 |
| HR Challenges | 2 | 2 |
| Critical skills shortages | 8 | 20 |
| High staff turnover | 1 | 3 |
| Lack of posts or jobs | 4 | 13 |
| Lacking Academic Platform | 1 | 5 |
| Non-engaged stakeholders | 1 | 1 |
| Skills deficit | 7 | 17 |
| Skills loss, e.g. brain drain | 3 | 4 |
| Maldistribution | 6 | 12 |
| Peri-urban based health facilities | 1 | 3 |
| Urban-based health facilities | 2 | 9 |
| Rurality | 7 | 27 |
| Economic growth and development | 1 | 1 |
| Social responsiveness | 10 | 40 |
| Governance and institutional development | 4 | 5 |
| Bursary Committee | 7 | 28 |
| Organisational structure (2) | 2 | 3 |
| Transformation | 2 | 3 |
| ROS structure | 4 | 6 |
| External Beneficiary | 8 | 104 |
| Funding model | 6 | 14 |
| Capped loan | 2 | 4 |
| Invoice-based Grant | 4 | 9 |
| Opt-out | 7 | 15 |
| ROS Advertisements | 6 | 18 |
| ROS Cost | 2 | 5 |
| Types of bursaries | 1 | 4 |
| Work Based Skills Plan | 6 | 8 |
| Strategic oversight | 0 | 0 |
| Beneficiary selection | 9 | 108 |
| Academic merit | 4 | 6 |
| Driven by interests of applicants | 1 | 1 |
| Other considerations | 0 | 0 |
| Equity across regions | 3 | 10 |
| Equity issues - particular groups selected (ethnicity, gender, etc.) | 4 | 8 |
| Program driven by the needs of the system | 1 | 4 |
| How is the total amount of money made available to the system determined | 0 | 0 |
| National versus provincial government priorities | 0 | 0 |
| Number of recipients | 10 | 76 |
| Type of health professionals trained | 4 | 11 |
| Oversight experience | 9 | 24 |
| Policy Evolution | 11 | 96 |
| Collaborations | 10 | 76 |
| Institutional memory | 7 | 33 |
| Policy Custodians | 9 | 26 |
| Policy Development | 6 | 29 |
| Policy development consultation | 5 | 11 |
| Policy document | 5 | 14 |
| Policy Framework | 8 | 37 |
| Policy genesis | 10 | 36 |
| Review | 9 | 44 |
| Solutions | 5 | 10 |
| Hopelessness | 6 | 13 |
| Integrity | 2 | 6 |
| Interoperable ROS Information System | 7 | 31 |
| Prospective Service Placement | 10 | 30 |
| Repetition and reenforcement | 3 | 3 |
| Regulatory body linkage | 8 | 27 |
| Local medical school | 2 | 2 |
| Restricted Internship | 8 | 20 |
| Opportunity costs of restricted internship | 2 | 2 |
| Rural recruitment advantages | 2 | 5 |
| Task shifting | 1 | 3 |
| Sustainability | 11 | 28 |
