# Supplementary material for: Are return-of-service bursaries an effective investment to build health workforce capacity? A qualitative study of key South African policymakers
Source: PLOS Glob Public Health. 2022 May 5;2(5):e0000309. doi: 10.1371/journal.pgph.0000309 (PMC10021585; doi:10.1371/journal.pgph.0000309)
Supplement: S1 Interview guide — (DOCX) [file pgph.0000309.s003.docx]

**The use of state sponsored educational initiatives for Sustainable Health Workforce Solutions: Alignment of Policies and Practices in Selected Southern African Countries**

**Individual or Group Interview sheet and interview guide for return-of-service scheme policymakers and/or policy-implementers**

Thank you so much for agreeing to take time from your busy schedule to answer a few questions on my research. I am doing a research study to find out more about the government policies used to fund health professionals in-training, in exchange for a period of service in the public health sector. The study aims to understand more about the history and evolution of these policies, how they relate with other human resources for health policies, their rationale, and how they are monitored and reviewed.

You are being interviewed because you are a manager that is involved in some way with the development and/or administration of policies that inform government sponsored bursaries or scholarships for health sciences students studying in the country or in other countries.

A group interview allows for a detailed discussion with a diverse group from the different units and divisions at once instead of hosting multitude of interviews with individuals within the department. That is the main reason why I have asked you to participate in the group interview. (For those who are unable to participate in a group interview this will read: I understand that it wasn’t possible for you to be part of a group interview due to your schedule. Because I value your contribution it is for this reason that I still requested to have an individual discussion with you). Please do not be intimidated by anyone as the information collected will only be used to enrich the schemes. All the names from this discussion will be de-identified and your identified responses will not be shared outside the research team. Your individual and diverse inputs are therefore highly valued. The aim of the research is not to assess professional competence and the outcomes of the research will not have a negative impact on your employment. In addition, you are welcome to refer to internal human resources and/or bursary/scholarship scheme related documents or even consult colleagues who you think might help remind you of detail that you might have forgotten. It’s also ok to not have all the answers. Please remember that the session is being audio recorded. You are welcome to let me know if you are not comfortable with that.

If you are happy with contents of this document and agree with the process could you kindly sign the consent forms and return to me before we start, if you haven’t already done so. I am happy to answer any questions that you may have before we begin the discussion. Are there any questions that any of you would like to ask on the process before we start?

| **No.** | **Area of Interest/topic** | **Initial broad descriptive questions** | **Possible probing questions** |
| --- | --- | --- | --- |
| 1 | Origins and evolution of the policy | - What is the departmental policy on bursaries for health sciences students? - What are the policy objectives? - In your understanding and knowledge, what has influenced the bursary policy for health sciences students? - As far as you know, when was this policy first introduced? - Could you enlighten me more about the development process and implementation of the bursary policy? - Which countries do beneficiaries of the policy go to for their studies? | - Could you please tell me more about any policy development frameworks used for developing your bursary policy or any other human resources for health policies? |
| 2 | Custodian of the policy | - Could you let me know which department or departments is or are responsible for the development and implementation of the bursary policy? - Could you give more information about the role of any other departments, offices or sections that could be involved? - Who makes the final decision on who receives an offer? | - How long has the situation been that way? - How has the process evolved over time |
| 3 | Review of the policy | - Is the bursary policy regularly reviewed? - What informs the reviewing of this policy? | - Is this related to political term? - What informs the need to review this policy? |
| 4 | Decision process | Could you tell me more about the process that informs the number of beneficiaries that can be funded in any particular funding cycle?  How are the opportunities advertised?  Can you tell me more about the selection criteria used to then select beneficiaries? | - How do you decide between the various categories of health sciences students that you fund? |
| 5 | Contract | In your view, what are the responsibilities of bursary recipients?  At what stage of the bursary offer do beneficiaries sign their contract?  What are the key contents of the contract?  What happens if a beneficiary defaults their contract? | - Is there an opt-out clause to the scheme? Elaborate… - What happens if a beneficiary doesn’t complete their studies? |
| 6 | Process after the completion of studies | How are the new graduates recruited into the health system?  At what stage of the process do you decide on the facility where the recipient will be placed in the health system?  What processes are used to identify the types of facilities that need the placement of beneficiaries?  At what stage do you plan for the salaries of beneficiaries? | - Are beneficiaries placed based on their own choices or on facilities chosen by government? - Who decides on the placement of graduates who previously benefited from the bursary scheme? |
| 7 | Policy Challenges | - What challenges has the policy encountered over the years? | - To your knowledge, have recipients defaulted their contracts previously? - What could be the reasons for beneficiaries to default bursary contracts? - What is the sustainability of the policy? |
| 8 | Monitoring and evaluation of the policy | - What processes are in place to ensure that beneficiaries fulfil their contractual obligations? - How often or uniformly are penalties imposed on those who default their contracts? - What features help or hinder monitoring of the program? - In your view, does the policy fulfil its objective? - If there is anything that you could change in the policy what would it be? - In your view, what are the ways that could have helped eliminate defaulting of the scheme? | - What Information systems are in place to monitor fulfilment of the policy? |

**Wrap-up**

Are the any other issues not covered that you would like to talk about?

**Thank you once more for your assistance, could you please also help me with a few documents that will help broaden my understanding on the schemes. You could add any other documents to the list if you think it or they will be of importance.**

| **List of documents to be requested** |
| --- |
| 1. All versions of bursary policy (current and historical) that are used to fund skilled health professionals. |
| 1. Blank copies of bursary contracts. |
| 1. Total health budget for the period 2000 to 2020. |
| 1. Health sciences bursary budget for the period 2000 to 2020. |
| 1. The total number of skilled health professionals (stratified by category and health facility) on 01 July 2020. |
| 1. The total number of skilled health professionals who are bursary beneficiaries (stratified by category and health facility) employed in the contracted government service area on 01 July 2020. |
| 1. Annual performance plans for the period 01 April 2015 to 31 March 2020. |
| 1. Annual performance reports for the period 01 April 2016 to 31 March 2021. |
